# Effects of Polyunsaturated Fatty Acids on Nonspecific Typical Dry Eye Disease: A Systematic Review and Meta-Analysis of Randomized Clinical Trials

**DOI:** 10.3390/nu11050942

**Published:** 2019-04-26

**Authors:** Sheng-Chu Chi, Hsin-I Tuan, Yi-No Kang

**Affiliations:** 1 Department of Ophthalmology, Taipei Veterans General Hospital, Taipei 110, Taiwan; b101100033@tmu.edu.tw; 2 Department of Ophthalmology, Chang Gung Memorial Hospital, Linkou, Taoyuan City 333, Taiwan; 3 Department of Medicine, Taipei Veterans General Hospital, Taipei 112, Taiwan; eosalvina@gmail.com; 4 School of Medicine, College of Medicine, Taipei Medical University, Taipei 110, Taiwan; 5 Center for Evidence-Based Medicine, Department of Education, Taipei Medical University Hospital, Taipei 110, Taiwan

**Keywords:** dry eye disease, polyunsaturated fatty acid, omega-3, omega-6

## Abstract

To investigate the effects of polyunsaturated fatty acids (PUFAs) in patients with dry eye disease (DED), a multifactorial inflammatory disorder, we searched Cochrane Library, EMBASE, PubMed, and Web of Science for randomized clinical trials (RCTs) investigating the effect of PUFAs in patients with DED before March 2019. Two reviewers independently abstracted data of tear breakup time (TBUT), Schirmer’s test, osmolarity, and ocular surface disease index (OSDI). We conducted pairwise meta-analysis using means and standard deviations (SDs) in a random-effects model for continuous outcomes. Thirteen eligible RCTs with 1782 patients with nonspecific typical DED were included. Patients who received PUFA treatment without other eye medications exhibited greater improvements in TBUT (MD = 1.80; *p* = 0.001), Schirmer test scores (MD = 0.50; *p* < 0.001), osmolarity (MD = −15.95; *p* < 0.001), and OSDI scores (MD = −10.19; *p* < 0.001) than those who received placebo treatment. However, the effects of PUFAs on TBUT (*p* < 0.001) and OSDI scores (*p* = 0.03) weakened with treatment duration. PUFAs are effective in treating nonspecific typical DED, particularly as a short-term treatment, with relatively few adverse events. Therefore, in real-world clinical practice, PUFA supplements are worth being suggested to patients with nonspecific typical DED who are not concurrently using other topical or systematic eye medications.

## 1. Introduction

Dry eye disease (DED) has long been recognized as a multifactorial inflammatory disorder [1], and refined by the Tear Film and Ocular Surface Society Dry Eye Workshop (TFOS DEWS) [2]. The 2017 update (TFOS DEWS-II) revised the DED definition and its classification under a pathophysiology-center scheme, highlighting the etiological continuum between the aqueous-deficient and evaporative dry eye. These two types of DED cause various symptoms that impair the quality of life [3,4], and staged therapy was recommended for DED management [5].

Current strategies DED management include enhancing tear volume and quality, reducing ocular inflammation, treating underlying lid disease, together with diet and lifestyle modifications [6]. As a dietary supplement, the essential fatty acids have been considered a promising supplementary treatment for there was clinical evidence showing inhibitory effects on inflammatory cytokines and T-cell responses [7,8]. An animal model study reported that polyunsaturated fatty acid (PUFA) could modify the phospholipid composition of the lacrimal gland and partially inhibit the local inflammations [9]. Furthermore, PUFAs increased lacrimal production by increasing lacrimal peroxidase activities [10].

In human observational studies, PUFAs appear to be a possible treatment for DED. In the United States, a women’s health study reported that diets containing high dose omega-3 fatty acid are negatively associated with dry eye symptoms [11]. Another study showed that omega-3 fatty acids plus artificial tears effectively alleviated dry eye symptoms [12]. Many randomized clinical trials (RCTs) evaluated tear film breakup time (TBUT), Schirmer test scores, and osmolarity, and ocular surface disease index (OSDI) score (subjective outcome) for understanding the effects of PUFA on DED. However, their results are heterogeneous. Some RCTs indicated that PUFAs significantly alleviated dry eye symptoms, and found improvement in both tear quality and quantity [13,14,15,16,17,18,19,20,21]. Nevertheless, other trials showed that PUFAs did not significantly improve TBUT and Schirmer test scores [22,23,24,25]. A big trial (the DREAM trial) recently reported PUFAs as having no benefit for patients with DED [22]. The effects of PUFAs on DED remain controversial.

The DREAM trial results mentioned that the patient selection, other treatments restriction, and duration of treatment may contribute to heterogeneities and controversies [22,26]. These factors possibly cause the heterogeneity in the outcomes among the previous RCTs. To our knowledge, no synthesized evidence stratifies PUFA alone and PUFA plus other eye treatments. Therefore, our primary aim was to examine the effects of PUFAs on DED by distinguishing PUFAs with and without other eye medications. Our study further tested the importance of duration of treatment on this topic. We focused on nonspecific typical DED because they are the general population in the real world.

## 2. Methods

We reported our study according to the Preferred Reporting Items for Systematic Reviews and Meta-Analyses guidelines. This study analyzing published data was exempted from institutional review board approval. Our study team registered the primary protocol on the PROSPERO with Registration number CRD42018109057.

### 2.1. Study Selection Criteria

According to the objective of our systematic review, we included studies if they met the following criteria: (1) the study recruited patients with DED, (2) the intervention was PUFA, and (3) the study design was RCT. The exclusion criteria were as follows: (1) gray literature did not report details, (2) the trial including both DED and other ocular diseases, (3) and studies only investigating specific DED. The specific DED study refers to the study recruiting patients with DED caused by a specific etiology. For instance, DED was caused by a specific disease, surgery, or patients’ behavior. As we know, the PUFA effects upon the DED were various in etiology [27], and non-specific DED was most similar to real world populations [22,26]. The effect of PUFA on specific DED should be separated from this synthesis in the future. 

### 2.2. Search Strategy and Study Selection

We performed electronic database search, without language and publication date restrictions, of the Cochrane Library, EMBASE, PubMed, and Web of Science databases from database inception until March 2019. We used the relevant search terms, the free text, and medical subject heading with appropriate Boolean functions. The detail searching strategy was recorded in the supplemental material. (Supplemental Material 1) Two reviewers (SCC and YNK) independently reviewed the returned references, and selected evidence according to eligible criteria. The screening and reviewing phases included removing duplications, title and abstract screen, and full-text review. The full texts of relevant articles were obtained and read by the authors.

### 2.3. Quality Assessment and Data Extraction

Two reviewers (SCC and YNK) independently assessed the methodological bias in each included study by using the Cochrane risk of bias tool. Three aspects and seven items associated with a risk of bias, namely random sequence generation, allocation concealment, blinding of participants and personnel, blinding of assessment, incomplete outcome data, selective reporting, and other sources of bias, were evaluated. In case of any disagreements on quality assessment between the two reviewers, a third reviewer (HIT) resolved the disagreement through discussion.

After quality assessment, the two reviewers (SCC and YNK) independently extracted relevant information and outcome data. The outcome data included three subjective symptom parameters, namely TBUT, Schirmer test scores, and osmolarity, and one objective symptom parameter, namely OSDI scores. In the RCT by Deinema et al., we extracted only the OSDI data of the krill-oil group because the OSDI data of the fish-oil group were incomplete. All of the aforementioned data were continuous, extracted as the means and their standard deviations (SDs). When RCTs presented standard errors (SEs), we estimated the SD by using the sample size according to relevant formula (SE = SD/√N). We not only identified and extracted the data but also double checked them independently.

### 2.4. Evidence Synthesis and Statistical Analysis

The included RCTs used both qualitative and quantitative approaches. An obvious cause of conceptual heterogeneity was baseline treatment. Therefore, we applied the random-effects model in our meta-analysis and further performed subset analysis for baseline treatment using dry eye medications. Thus, we separated “other eye medications were excluded” and “other eye medications were continued”. The “other eye medications were continued” referred to other medications plus PUFA versus other medications because trials usually set the same rule on the usage of baseline treatment for both PUFA groups and control groups. For studies using a quantitative approach, we conducted pairwise meta-analysis by using the means and SDs for continuous outcomes. When an outcome was measured using the same scale, the mean differences (MDs) and SDs were determined using 95% confidence intervals (CIs) as well as I^2^ values. When 95% CIs did not cross the cutoff point of 0, the outcome was considered significantly different. When I^2^ was >50%, the outcome was considered highly heterogeneous. If a result showed high heterogeneity, we tried to detect the source of variance through meta-regression. In meta-regression, intervention duration (month) served as a predictor. To detect small-study bias, we constructed a funnel plot and performed the Egger test. All pairwise meta-analyses were completed using RevMan (version 5.3 for Microsoft Windows, Copenhagen, Denmark). The results of meta-regression and the Egger test were analyzed in the Comprehensive Meta-Analysis (version 2 for Microsoft Windows, Biostat, Inglewood, NJ, USA).

## 3. Results

**Figure 1:**
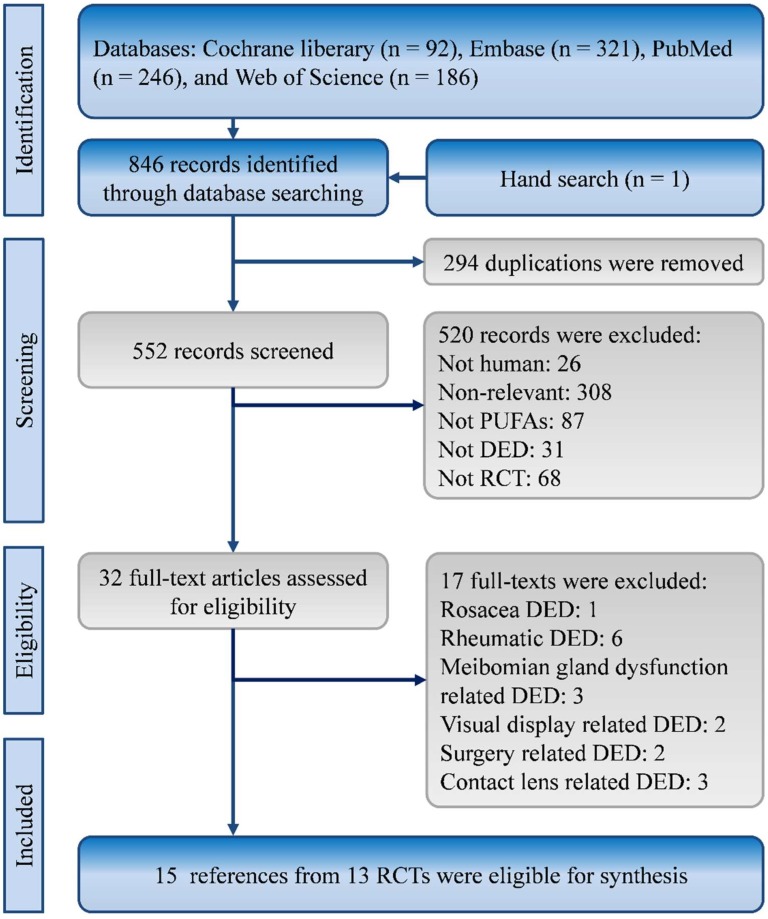
Flowchart of study selection.

In total, 846 potential studies were identified from the Cochrane Library (*n* = 92), EMBASE (*n* = 321), PubMed (*n* = 246), and Web of Science (*n* = 186). One record was returned from a hand search of reference lists. After the exclusion of 294 duplicate articles, 552 articles were assessed for eligibility. Finally, 15 records from 13 RCTs met the eligibility criteria [13,14,15,16,17,18,19,21,22,23,24,25,26,28]. The process of evidence selection is shown in Figure 1.

### 3.1. Characteristics and Quality of Included Studies

The characteristics of included studies are listed in Table 1. The 13 eligible RCTs were published between 2003 and 2018. In total, these RCTs recruited 1782 patients with nonspecific typical DED. The mean ages of each group ranged from 38.82 to 63.4 years. Most patients were female (*n* = 1314; 73.6%). A total of seven trials used low dose eicosapentaenoic acid (EPA) and docosahexaenoic acid (DHA) lower than 1000 mg per day [14,16,18,21,23,25,28], and the other four trials used high dose intervention by giving EPA and DHA more than 1000 mg per day [15,17,19,22]. There were only two trials that did not use EPA or DHA. One of the two trials used α-Linolenic acid (ALA, 149 mg per day) and Linoleic acid (LA, 245 mg) per day [24], and the other one provided patients gamma-Linolenic acid (GLA, 30 mg) 30 and LA (57 mg) per day [13]. Among the 11 trials using EPA and DHA, four of them also provided patients GLA [16,21,23,28]. The GLA was usually given within 100 mg per day [13,16,23,28], and there was only one trial providing GLA 240 mg per day [21]. The details of PUFA in each trial can be found in Supplemental Material 2. Four of the 13 RCTs allowed the patients to use other eye medications during the trial [16,22,23,24], whereas the other trials suspended other eye medications. Three trials reported the use of anesthesia when conducting the Schirmer test [14,17,22]. The follow-up durations of the 13 RCTs varied from one month to 12 months. The quality of eligible trials is presented in Supplemental Material 3.

### 3.2. TBUT Improvement

**Figure 2:**
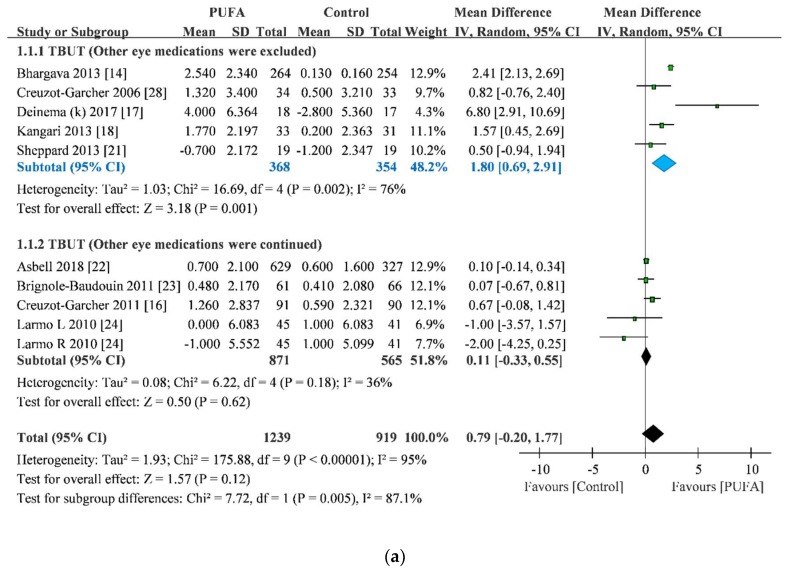

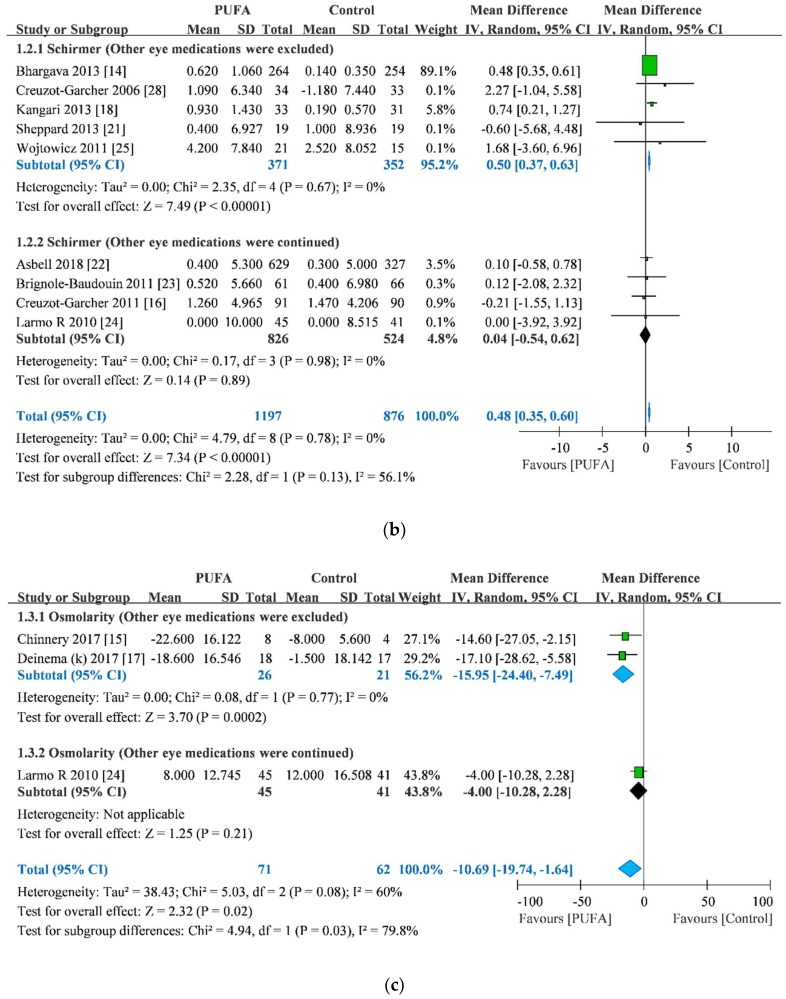

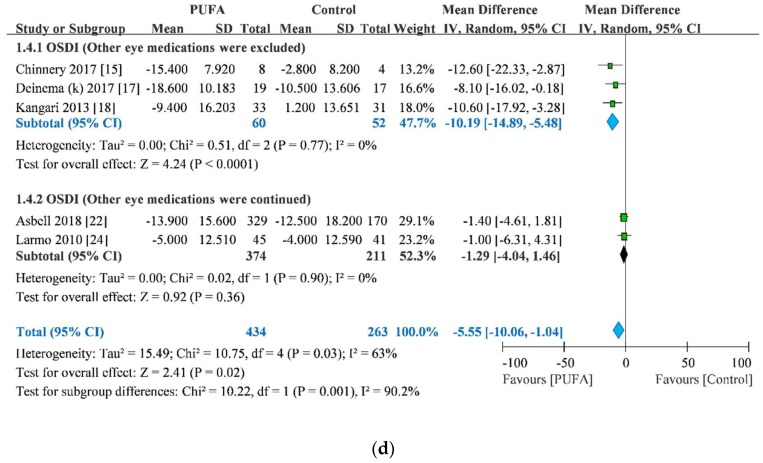
Forest plot of tear breakup time (**a**), Schirmer’s test score (**b**), osmolarity (**c**), and ocular surface disease index score (**d**). Blue part: statistical significance.

Nine of the RCTs reported improvement in TBUT [14,16,17,18,21,22,23,24,28]. One of them, by Larmo et al., separately reported data from both the eyes [24]. The pooled result of TBUT improvement indicated that the PUFA group exhibited a greater improvement in TBUT than the control group (MD = 0.79, 95% CI = −0.20 to 1.77), but this overall pooling estimate was not significant (*p* = 0.12) with very high heterogeneity (I^2^ = 95%). In subset analysis, interestingly, the PUFA group exhibited a greater improvement in TBUT than the control group when other eye medications were suspended (MD = 1.80, 95% CI = 0.69 to 2.91; *p* = 0.001). The heterogeneity was slightly reduced (I^2^ = 76%), but it was still high. By contrast, when other eye medications were allowed in DED management, the PUFA group showed nonsignificant TBUT improvement compared with the control group (MD = 0.11, 95% CI = −0.33 to 0.55; *p* = 0.62; Figure 2). Because the subset analysis still showed high heterogeneity, we applied sensitivity analysis to explore individual study effects on overall pooling. The sensitivity analysis showed that the pooled result was not affected when any one study was removed (Supplemental Material 4). Obviously, the trial by Deinema et al. contributed to the heterogeneity according to the forest plot, but the sensitivity demonstrated that PUFA group still owned a greater improvement in TBUT than the control group after the trial was excluded (MD = 1.50, 95% CI = −0.51 to 2.50). No evidence showed a small-study effect on this result (Egger test = −0.53; *p* = 0.81) (Supplemental Material 5). 

Additional analysis showed that the pooled results of TBUT improvement were not associated with data from a single eye or from a mean of a pair of eyes but were associated with treatment duration (point estimate = −0.20; *p* < 0.001). Moreover, the meta-regression of treatment duration on TBUT improvement reduced variance (τ^2^) from 1.90 to 1.62 (Table 2 and Supplemental Materials 6,7).

### 3.3. Schirmer Test Score Improvement

Nine of the 13 RCTs presented improvements in Schirmer test scores [14,16,18,21,22,23,24,25,28]. The trial by Larmo et al. only reported the data of the improvement in Schirmer test scores of the right eye [24]. The pooled result showed that the PUFA group exhibited significantly greater improvements in Schirmer test score, with low heterogeneity, than the control group (MD = 0.48, 95% CI = 0.35 to 0.60; *p* < 0.001; I^2^ = 0%). Notably, subset analysis results revealed that the PUFA group showed greater improvement in Schirmer test score than the control group when other eye medications were suspended (MD = 0.50, 95% CI = 0.37 to 0.63; *p* < 0.001); however, the PUFA group exhibited nonsignificant improvements in Schirmer test score compared with the control group when the use of other eye medications was allowed (MD = 0.04, 95% CI = −0.54 to 0.62; *p* = 0.89; Figure 2). We did not detect a small-study effect on this result (Egger test = −0.08; *p* = 0.81) (Supplemental Material 8).

The improvement in Schirmer test score was not affected either by single-eye data or by treatment duration (Table 2). Because some trials reported the use of anesthesia while conducting the Schirmer test, the result of the improvement in Schirmer test score were tested using meta-regression of anesthesia use. The meta-regression showed that the improvement in Schirmer test score was not associated with anesthesia use (Table 2 and Supplemental Materials 9–11).

### 3.4. Osmolarity Improvement

Three of the RCTs reported improvement in osmolarity [15,17,24]. The RCT by Larmo et al. only reported the data of osmolarity improvement in the right eye [24]. The pooled results showed that the PUFA group exhibited a significantly lower osmolarity, with high heterogeneity, than the control group (MD = −10.69, 95% CI = −19.74 to −1.64; *p* = 0.02; I^2^ = 60%). In subset analysis, the PUFA group without other eye medications exhibited a significantly lower osmolarity than the control group (MD = −15.95, 95% CI = −24.40 to −7.49; *p* < 0.001); however, the PUFA group did not exhibit a significantly lower osmolarity than the control group in the subset that allowed the use of other eye medications (MD = −4.00, 95% CI = −10.28 to 2.28; *p* = 0.21; Figure 2). No evidence of the small-study effect was observed in this result (Egger test = −2.83; *p* = 0.32) (Supplemental Material 12). Because there were only three trials reporting the result of osmolarity, the present meta-analysis cannot examine the association between osmolarity improvement and either single-eye data or treatment duration.

### 3.5. OSDI Score Improvement

In total, five RCTs reported improvements in OSDI scores [15,17,18,22,24]. The pooled results showed that the PUFA group exhibited significantly lower OSDI score with high heterogeneity than did the control group (MD = −5.55, 95% CI = −10.06 to −1.04; *p* = 0.02; I^2^ = 63%). In subset analysis, which suspended other eye medications, the PUFA group exhibited significantly lower OSDI score than the control group (MD = −10.19, 95% CI = −14.89 to −5.48; *p* < 0.001). By contrast, the PUFA group did not exhibit significantly lower OSDI score than the control group in the subset that allowed the use of other eye medications (MD = −1.29, 95% CI = −4.04 to 1.46; *p* = 0.36; Figure 2). No evidence of the small-study effect was observed in this result (Egger test = −3.18; *p* = 0.05; Begg and Mazumdar rank correlation = −0.50; *p* = 0.22) (Supplemental Material 13).

Additional analysis showed that the pooled results of OSDI score improvement were not affected by either data from a single eye or data from a mean of pair of eyes. Nevertheless, it was modified by treatment duration (point estimate = 0.54; *p* = 0.03). Moreover, the meta-regression of treatment duration on OSDI score improvement slightly reduced τ^2^ from 15.83 to 15.27 (Table 2 and Supplemental Material 14).

## 4. Discussion

### 4.1. Key Findings

This systematic review and meta-analysis consisted of 13 RCTs involving 1782 patients with nonspecific typical DED. The overall results showed that PUFA without other eye medications significantly improved TBUT, Schirmer test score, osmolarity, and OSDI score. By contrast, PUFA supplements concurrent with other eye treatments did not improve these outcomes in the patients with DED. Our pooled finding about the effect of PUFA on DED is consistent with the other two included RCTs though these two trials did not present available data for quantitative synthesis [13,19]. These two RCTs concluded that PUFA is recommended for treating patients with DED, and the conclusions were based on significant outcomes. Interestingly, one of the two trials also performed both TBUT and the Schirmer test, and the trial reported that PUFA had greater TBUT improvement than control while there was no significant difference in Schirmer test between two groups [19]. Although these findings were not available for our meta-analysis, we found similar results in our pooled analyses. Furthermore, in our pooled results, treatment duration was associated with the TBUT improvement and OSDI score. These results demonstrated that PUFA exerted a short-term beneficial effect on TBUT and OSDI score. Thus, different treatment durations of the included RCTs resulted in heterogeneity in improvements in TBUT and OSDI score.

In response to the DREAM trial, our study only included RCTs that recruited patients with nonspecific typical DED. Because the DREAM trial was designed to reflect the real-world situation in DED management, it had minimally restrictive eligibility criteria to recruit a broad spectrum of typical DED patients [22,26]. These patients were extremely similar to the patients most commonly observed in real-world clinical practice. Similarly, our study included RCTs recruiting patients with nonspecific typical DED. However, our results differed from that of the DREAM trial. We found that the PUFA group exhibited significant improvements in Schirmer test score, osmolarity, and OSDI scores compared with the control group. Nevertheless, high heterogeneity was noted in the TBUT (I^2^ = 95%), osmolarity (I^2^ = 60%), and OSDI score (I^2^ = 63%).

To explore the sources of heterogeneities in our outcomes, we stratified studies by whether the other eye medication was excluded in their eligible criteria. Consequently, we observed relatively low heterogeneities in all subset analyses. Most heterogeneities were reduced to very low levels in subset analysis, except for TBUT. Moreover, we observed high to very high heterogeneities between subsets. TBUT (I^2^ = 87.1%), Schirmer test score (I^2^ = 56.1%), osmolarity (I^2^ = 79.8%), and OSDI scores (I^2^ = 90.2%). Nevertheless, high heterogeneity (I^2^ = 76%) was observed in the subset of TBUT when other eye medications were suspended. Thus, in our study, we attempted to understand this heterogeneity and put treatment duration in our meta-regression study to explore the association between treatment duration and outcomes. Consequently, our study successfully identified a negative correlation between treatment duration and two outcomes, namely improvements in TBUT and OSDI score. In addition, the results showed a decrease in τ^2^ values in these two outcomes.

Nevertheless, we cannot deny that many factors relate to the high heterogeneity in the subset of TBUT because tear breakup is a complicated process and is affected by multiple factors [29]. In this complicated process, the lipid layer in the tear film is crucial in preventing the tear film from collapsing [30]. An increase in tear volume makes the tear film thicker and prolongs TBUT [31]. Although PUFAs, a type of lipid, may not prevent underlying disease progress, they may improve tear quality and quantity in tear breakup process. Thus, the DED-attenuating effects of PUFAs in our study may be due to the role of PUFAs in curing DED. Moreover, trial design, characteristics of patients, and composition of PUFAs supplement are also factors causing heterogeneity. For instance, in the high heterogeneity subset analysis of TBUT, the effect size of the trial by Deinema et al. was far from other trials, and might contribute to heterogeneity. Actually, the data of PUFA group in the trial by Deinema et al. was based on krill oil arm. As we know, the krill oil involving EPA and DHA in phospholipid form is different from fish oil, and its bioavailability is better than fish oil [32]. In addition, krill oil contains antioxidant astaxanthin, which made it more stable [33]. Deinema et al. also indicated that the krill oil decreased IL-17 in tears significantly [17]. The role of composition and posology of PUFAs in curing DED should be surveyed in further study.

Regarding the safety of PUFAs, few severe adverse events have been reported in the RCTs we included in this systematic review. Most of the adverse events were gastric intolerance. The DREAM trial used a protocol of the highest dosage, omega-3 (3000 mg daily), and the trial exhibited no significant difference in adverse events between intervention group and control group [22,26]. Most patients appeared to tolerate PUFAs adequately well.

### 4.2. Comparison with the Largest Trial

The DREAM trial, the largest trial on this topic, argued that the study designs in the previous RCTs were not applicable to the real world, and that broad recruitment of patients with DED was required. We also noted that some RCTs allowing other topical or systematic DED treatments found similar results [22,24]. By contrast, the other trials excluding other eye medications had opposite results [14,17,18]. We completely agree with the DREAM trial design according to the real-world situation. However, the results of the DREAM trial can be only interpreted as no benefits in moderate to severe DED from PUFA when it was an adjuvant treatment. The real effects of PUFA should be objectively proved by pure data. 

The effects of PUFA itself on DED should be interpreted according to pure data without other eye medications. For instance, significant improvements can be concurrently observed in both intervention and control groups because of other DED medications. Some eye medications have demonstrated effective therapeutic effects in patients with DED. Several RCTs reported that topical glucocorticoid can relieve DED symptoms and the inflammation of the ocular surface [34,35,36]. A recent meta-analysis also reported that topical cyclosporine is a promising treatment for DED and can improve TBUT, Schirmer test score, corneal staining results, and OSDI score [37]. In the DREAM trial, there were about 38% of patients using the cyclosporine drop and 50% of patients using other treatments. That is to say, the results in the DREAM trial may be affected by other eye treatments. In contrast, our study provided an overview for the effects of PUFAs on DED by concurrently showing with and without other eye medications. 

### 4.3. Comparison with Previous Synthesized Evidence

Two synthesized studies were published before our study. One is a meta-analysis in 2014 and the other is systematic review in 2017 [27,38]. The previous meta-analysis included only nine RCTs with different DED etiologies, some of which included recruited patients with only rheumatoid DED [38]. The pooled results from different etiologies may have affected the effects of PUFAs on DED [27]. Although the meta-analysis found that PUFAs improved OSDI score, the result was highly heterogenous (I^2^ = 66%) [38]. Moreover, the meta-analysis presented incorrect data of OSDI scores in the study by Larmo et al. [24,38]. In addition, the RCTs by Barabino et al. were pooled in the analysis of OSDI scores in the meta-analysis [13,38], but the subjective outcome of Barabino et al. was not the OSDI score (Rolando score system) [13].

Another piece of synthesized evidence, a systematic review without meta-analysis, presented clear and rigorous synthesis on the topic of PUFAs for DED management [27]. The systematic review identified 15 RCTs on the topic PUFAs for DED management published between 2005 and 2015 and categorized the trials by etiology of DED. The systematic review clearly demonstrated the effects of PUFAs in DED with different etiology. However, the study only concluded that the available evidence showed variable results on the effects of PUFAs for DED and suggested that omega-3 should be the main component of oral supplements [27]. In our study, although we included patients with nonspecific typical DED, our systematic review involved the latest evidence and clarified the controversy regarding the effects of PUFAs for DED through meta-analysis. Therefore, our evidence may provide further appropriate suggestions for clinical practice.

### 4.4. Limitations and Future Direction

Our study has numerous limitations. First, the RCTs we included exhibited variation in patient characteristics, such as age, sex, dietary practices, and DED severity. Because the RCTs provided different data but not extensively, we could not perform an in-depth investigation on the specific variations. Thus, individual patient data are needed for future meta-analysis. Second, the conceptual heterogeneity in this systematic review was present in not only the study populations but also the interventions. These RCTs implemented different types and doses of PUFAs, baseline treatments, and treatment durations. We have tried our best to reduce some of the variation in baseline treatment types and treatment durations through subset analysis and meta-regression. The results showed reduction in heterogeneity in further analysis. However, the limited number of evidences in our synthesis may affect the robustness in statistics. For example, some subset analysis pooled fewer than three studies, and the meta-regression pooled fewer than ten studies. We conceded meta-regression need an appropriately large ratio of studies to investigate other covariates. These analyses may give some hints for researchers and clinicians in future study and clinical practice. Third, because most of the studies did not review patient compliance by testing serum fatty acid levels objectively, we cannot guarantee compliance by all the patients in all the RCTs. Patient compliance is crucial in outpatients with medications or supplement therapy. Finally, few studies have reported the complete data of each measurement during the treatment course; most studies have only reported outcomes at the endpoint. We explored the trends of treatment durations on PUFA effects and found the meaningful results with regard to TBUT and OSDI score. However, the optimal treatment duration for patients with DED remains unknown. Therefore, an additional longitudinal study to optimize treatment duration when PUFAs is used as supplemental therapy for DED is warranted.

## 5. Conclusions

PUFAs without other eye medications effectively improved TBUT, Schirmer test score, osmolarity, and OSDI score in patients with nonspecific DED. Regarding improvements in TBUT, PUFAs may exhibit only short-term effects. Moreover, the studies included in the current systematic review have reported few adverse events in patients who received omega-3 or omega-6. Therefore, in the real-world clinical practice, it is worth suggesting PUFAs to patients with nonspecific topical DED if they are not concurrently using other topical or systematic treatments. Additional RCTs should explore the effects of duration of treatment, and determine the optimal dosage for PUFA treatment.

## Figures and Tables

**Table 1:** Characteristics of included studies.

Study	Year	Sample Size		Age		Sex (male/female)		Other Eye Medication	Treatment Duration	Anesthesia for Schirmer Test
		PUFA	Control	PUFA	Control	PUFA	Control			
Asbell et al. [22]	2018	349	186	58.3 ± 13.5	57.5 ± 12.6	65/284	36/150	Allowed	12 months	Yes
Barabino et al. [13]	2003	13	13	63.4 ± 8.2	54.3 ± 11.3	4/9	3/10	Limited	1.5months	NR
Bhargava et al. [14]	2013	264	254	38.82±4.12	40.06 ± 6.76	Total:	254/268	Limited	3 months	Yes
Brignole-Baudouin et al. [23]	2011	58	63	60 ± 11.75	59.7 ± 11.95	1/57	3/60	Allowed	3 months	NR
Chinnery et al. [15]	2017	8	4	42 ± 7 ^a^	46 ± 10 ^a^	2/6	1/3	Limited	3 months	NR
Creuzo-Garcher et al. [16]	2011	90	91	61.28 ± 12.15	61.79 ± 11.64	8/82	7/84	Allowed	6 months	NR
Creuzot-Garcher et al. [28]	2006	36	35	59.7 ± 14.7	61.1 ± 11.1	2/34	1/34	Limited	6 months	No
Deinema et al. [17]	2017	37	17	Total:	42.51	Total:	18/36	Limited	3 months	Yes
Kangari et al. [18]	2013	33	31	60.6 ± 8.7	61.8 ± 8	15/18	11/20	Limited	1 month	No
Kawakita et al. [19]	2013	15	11	52.5 ± 2.5 ^a^	51.9 ± 2.2 ^a^	5/10	1/10	Limited	4 months	No
Larmo et al. [24]	2010	52	48	45 ± 18	46 ± 17	8/44	7/41	Allowed	3 months	No
Sheppard et al. [21]	2013	19	19	62 ± 1 ^a^	61 ± 2 ^a^	0/19	0/19	Limited	6 months	NR
Wojtowicz et al. [25]	2011	21	15	Total:	61	Total:	20/16	Limited	3 months	No

^a^ Standard error. PUFA, polyunsaturated fatty acid. NR, not reported.

**Table 2:** Outcomes of small-study effect and meta-regression.

Outcome	Small-Study Effect		Meta-Regression by Duration			Meta-Regression by Single-eye Data			Meta-Regression by Type of Schirmer Test		
	Egger test	*P*	Point Estimate	*P*	*τ* ^2^	Point Estimate	*P*	*τ* ^2^	Point Estimate	*P*	*τ* ^2^
TBUT	−0.53	0.81	−0.20	<0.001	1.62	0.17	0.55	2.16	NE	NE	NE
Schirmer test scores	−0.08	0.81	−0.05	0.17	0.00	−0.17	0.52	0.00	−0.31	0.26	0.00
Osmolarity	−2.83	0.32	NE	NE	NE	NE	NE	NE	NE	NE	NE
OSDI score	−3.18	0.05	0.54	0.03	15.27	NE	NE	NE	NE	NE	NE

OSDI: ocular surface disease index. TBUT: tear breakup time. NE, not estimate.

## Supplementary Materials

The following are available online at https://www.mdpi.com/2072-6643/11/5/942/s1, Supplemental Material 1: Search strategy, Supplemental Material 2: Contents and dosage of polyunsaturated fatty acids, Supplemental Material 3: Risk of bias, Supplemental Material 4: Sensitivity analysis for high heterogeneity outcome (TBUT), Supplemental Material 5: Small study effect test (TBUT), Supplemental Material 6: Meta-regression of treatment duration on TBUT, Supplemental Material 7: Meta-regression of single-eye data on TBUT, Supplemental Material 8: Small study effect test (Schirmer’s test), Supplemental Material 9: Meta-regression of treatment duration on Schirmer’s test score, Supplemental Material 10: Meta-regression of single-eye data on Schirmer’s test score, Supplemental Material 11: Meta-regression of anesthesia on Schirmer’s test score, Supplemental Material 12: Small study effect test (Osmolarity), Supplemental Material 13: Small study effect test (OSDI score), Supplemental Material 14: Meta-regression of treatment duration on OSDI score.

## Abbreviations

ALA	α-linolenic acid
CI	confidence interval
DED	dry eye disease
DHA	docosahexaenoic acid
EPA	eicosapentaenoic acid
GLA	gamma-Linolenic acid
LA	linoleic acid
MD	mean difference
OSDI	ocular surface disease index
PUFA	polyunsaturated fatty acid
RCT	randomized clinical trial
SD	standard deviation
SE	standard error
TBUT	tear breakup time

## References

[1] Pflugfelder S.C., de Paiva C.S. (2017). The pathophysiology of dry eye disease: What we know and future directions for research. Ophthalmology.

[2] Herrero-Vanrell R., Peral A. (2007). International dry eye workshop (dews). Update of the disease. Arch. Soc. Esp. Oftalmol..

[3] Craig J.P., Nelson J.D., Azar D.T., Belmonte C., Bron A.J., Chauhan S.K., de Paiva C.S., Gomes J.A.P., Hammitt K.M., Jones L. (2017). Tfos dews ii report executive summary. Ocul. Surf..

[4] Craig J.P., Nichols K.K., Akpek E.K., Caffery B., Dua H.S., Joo C.-K., Liu Z., Nelson J.D., Nichols J.J., Tsubota K. (2017). Tfos dews ii definition and classification report. Ocul. Surf..

[5] Jones L., Downie L.E., Korb D., Benitez-Del-Castillo J.M., Dana R., Deng S.X., Dong P.N., Geerling G., Hida R.Y., Liu Y. (2017). Tfos dews ii management and therapy report. Ocul. Surf..

[6] Messmer E.M. (2015). The pathophysiology, diagnosis, and treatment of dry eye disease. Dtsch. Arztebl Int..

[7] Endres S., Ghorbani R., Kelley V.E., Georgilis K., Lonnemann G., van der Meer J.W., Cannon J.G., Rogers T.S., Klempner M.S., Weber P.C. (1989). The effect of dietary supplementation with n-3 polyunsaturated fatty acids on the synthesis of interleukin-1 and tumor necrosis factor by mononuclear cells. N. Engl. J. Med..

[8] Zurier R.B., Rossetti R.G., Seiler C.M., Laposata M. (1999). Human peripheral blood t lymphocyte proliferation after activation of the t cell receptor: Effects of unsaturated fatty acids. Prostaglandins Leukot. Essent. Fatty Acids.

[9] Viau S., Maire M.A., Pasquis B., Gregoire S., Acar N., Bron A.M., Bretillon L., Creuzot-Garcher C.P., Joffre C. (2009). Efficacy of a 2-month dietary supplementation with polyunsaturated fatty acids in dry eye induced by scopolamine in a rat model. Graefes. Arch. Clin. Exp. Ophthalmol..

[10] Andrade A.S., Salomon T.B., Behling C.S., Mahl C.D., Hackenhaar F.S., Putti J., Benfato M.S. (2014). Alpha-lipoic acid restores tear production in an animal model of dry eye. Exp. Eye Res..

[11] Miljanovic B., Trivedi K.A., Dana M.R., Gilbard J.P., Buring J.E., Schaumberg D.A. (2005). Relation between dietary n-3 and n-6 fatty acids and clinically diagnosed dry eye syndrome in women. Am. J. Clin. Nutr..

[12] Oleñik A. (2014). Effectiveness and tolerability of dietary supplementation with a combination of omega-3 polyunsaturated fatty acids and antioxidants in the treatment of dry eye symptoms: Results of a prospective study. Clin. Ophthalmol. (Auckland, N.Z.).

[13] Barabino S., Rolando M., Camicione P., Ravera G., Zanardi S., Giuffrida S., Calabria G. (2003). Systemic linoleic and gamma-linolenic acid therapy in dry eye syndrome with an inflammatory component. Cornea.

[14] Bhargava R., Kumar P., Kumar M., Mehra N., Mishra A. (2013). A randomized controlled trial of omega-3 fatty acids in dry eye syndrome. Int. J Ophthalmol..

[15] Chinnery H.R., Golborne C.N., Downie L.E. (2017). Omega-3 supplementation is neuroprotective to corneal nerves in dry eye disease: A pilot study. Ophthalmic Physiol. Opt..

[16] Creuzot-Garcher C., Baudouin C., Labetoulle M., Pisella P.J., Mouriaux F., Meddeb-Ouertani A., El Matri L., Khairallah M., Brignole-Baudouin F. (2011). Efficacy assessment of nutrilarm(r), a per os omega-3 and omega-6 polyunsaturated essential fatty acid dietary formulation versus placebo in patients with bilateral treated moderate dry eye syndrome. J. Fr. Ophtalmol..

[17] Deinema L.A., Vingrys A.J., Wong C.Y., Jackson D.C., Chinnery H.R., Downie L.E. (2017). A randomized, double-masked, placebo-controlled clinical trial of two forms of omega-3 supplements for treating dry eye disease. Ophthalmology.

[18] Kangari H., Eftekhari M.H., Sardari S., Hashemi H., Salamzadeh J., Ghassemi-Broumand M., Khabazkhoob M. (2013). Short-term consumption of oral omega-3 and dry eye syndrome. Ophthalmology.

[19] Kawakita T., Kawabata F., Tsuji T., Kawashima M., Shimmura S., Tsubota K. (2013). Effects of dietary supplementation with fish oil on dry eye syndrome subjects: Randomized controlled trial. Biomed. Res..

[20] Pinazo-Duran M.D., Galbis-Estrada C., Pons-Vazquez S., Cantu-Dibildox J., Marco-Ramirez C., Benitez-del-Castillo J. (2013). Effects of a nutraceutical formulation based on the combination of antioxidants and omega-3 essential fatty acids in the expression of inflammation and immune response mediators in tears from patients with dry eye disorders. Clin. Interv. Aging.

[21] Sheppard J.D., Singh R., McClellan A.J., Weikert M.P., Scoper S.V., Joly T.J., Whitley W.O., Kakkar E., Pflugfelder S.C. (2013). Long-term supplementation with n-6 and n-3 pufas improves moderate-to-severe keratoconjunctivitis sicca: A randomized double-blind clinical trial. Cornea.

[22] Asbell P.A., Maguire G., Pistilli M., Ying G.S., Szczotka-Flynn L.B., Hardten D.R., Lin M.C., Shtein R.M. (2018). N−3 fatty acid supplementation for the treatment of dry eye disease. N. Engl. J. Med..

[23] Brignole-Baudouin F., Baudouin C., Aragona P., Rolando M., Labetoulle M., Pisella P.J., Barabino S., Siou-Mermet R., Creuzot-Garcher C. (2011). A multicentre, double-masked, randomized, controlled trial assessing the effect of oral supplementation of omega-3 and omega-6 fatty acids on a conjunctival inflammatory marker in dry eye patients. Acta. Ophthalmol..

[24] Larmo P.S., Jarvinen R.L., Setala N.L., Yang B., Viitanen M.H., Engblom J.R., Tahvonen R.L., Kallio H.P. (2010). Oral sea buckthorn oil attenuates tear film osmolarity and symptoms in individuals with dry eye. J. Nutr..

[25] Wojtowicz J.C., Butovich I., Uchiyama E., Aronowicz J., Agee S., McCulley J.P. (2011). Pilot, prospective, randomized, double-masked, placebo-controlled clinical trial of an omega-3 supplement for dry eye. Cornea.

[26] Asbell P.A., Maguire M.G., Peskin E., Bunya V.Y., Kuklinski E.J. (2018). Dry eye assessment and management (dream(c)) study: Study design and baseline characteristics. Contemp. Clin. Trials.

[27] Molina-Leyva I., Molina-Leyva A., Bueno-Cavanillas A. (2017). Efficacy of nutritional supplementation with omega-3 and omega-6 fatty acids in dry eye syndrome: A systematic review of randomized clinical trials. Acta. Ophthalmol..

[28] Creuzot C., Passemard M., Viau S., Joffre C., Pouliquen P., Elena P.P., Bron A., Brignole F. (2006). Improvement of dry eye symptoms with polyunsaturated fatty acids. J. Fr. Ophtalmol..

[29] King-Smith P.E., Begley C.G., Braun R.J. (2018). Mechanisms, imaging and structure of tear film breakup. Ocul. Surf..

[30] Millar T.J., Schuett B.S. (2015). The real reason for having a meibomian lipid layer covering the outer surface of the tear film - a review. Exp. Eye Res..

[31] Wong H., Fatt I.I., Radke C.J. (1996). Deposition and thinning of the human tear film. J. Colloid. Interface Sci..

[32] Ulven S.M., Holven K.B. (2015). Comparison of bioavailability of krill oil versus fish oil and health effect. Vas. Health Risk Manag..

[33] Bustos R., Romo L., Yáñez K., Díaz G., Romo C. (2003). Oxidative stability of carotenoid pigments and polyunsaturated fatty acids in microparticulate diets containing krill oil for nutrition of marine fish larvae. J. Food Eng..

[34] Kheirkhah A., Dohlman T.H., Amparo F., Arnoldner M.A., Jamali A., Hamrah P., Dana R. (2015). Effects of corneal nerve density on the response to treatment in dry eye disease. Ophthalmology.

[35] Pinto-Fraga J., Lopez-Miguel A., Gonzalez-Garcia M.J., Fernandez I., Lopez-de-la-Rosa A., Enriquez-de-Salamanca A., Stern M.E., Calonge M. (2016). Topical fluorometholone protects the ocular surface of dry eye patients from desiccating stress: A randomized controlled clinical trial. Ophthalmology.

[36] Sheppard J.D., Donnenfeld E.D., Holland E.J., Slonim C.B., Solomon R., Solomon K.D., McDonald M.B., Perry H.D., Lane S.S., Pflugfelder S.C. (2014). Effect of loteprednol etabonate 0.5% on initiation of dry eye treatment with topical cyclosporine 0.05%. Eye Contact Lens.

[37] Wan K.H., Chen L.J., Young A.L. (2015). Efficacy and safety of topical 0.05% cyclosporine eye drops in the treatment of dry eye syndrome: A systematic review and meta-analysis. Ocul. Surf..

[38] Zhu W., Wu Y., Li G., Wang J., Li X. (2014). Efficacy of polyunsaturated fatty acids for dry eye syndrome: A meta-analysis of randomized controlled trials. Nutr. Rev..

