# Supplementary material for: Effects of Polyunsaturated Fatty Acids on Nonspecific Typical Dry Eye Disease: A Systematic Review and Meta-Analysis of Randomized Clinical Trials
Source: Nutrients. 2019 Apr 26;11(5):942. doi: 10.3390/nu11050942 (PMC6566338; doi:10.3390/nu11050942)
Supplement: Supplementary file 1 [file nutrients-11-00942-s001.pdf]

Effects of polyunsaturated fatty acids on nonspecific typical dry eye disease:

A systematic review and meta-analysis of randomized clinical trials

(Supplemental Materials)

Supplemental Material

Supplemental Material 1. Search strategy

Supplemental Material 2. Contents and dosage of polyunsaturated fatty acids

Supplemental Material 3. Risk of bias

Supplemental Material 4. Sensitivity analysis for high heterogeneity outcome (TBUT)

Supplemental Material 5. Small study effect test (TBUT)

Supplemental Material 6. Meta-regression of treatment duration on TBUT

Supplemental Material 7. Meta-regression of single-eye data on TBUT

Supplemental Material 8. Small study effect test (Schirmer's test)

Supplemental Material 9. Meta-regression of treatment duration on Schirmer's test score

Supplemental Material 10. Meta-regression of single-eye data on Schirmer's test score

Supplemental Material 11. Meta-regression of anesthesia on Schirmer's test score

Supplemental Material 12. Small study effect test (Osmolarity)

Supplemental Material 13. Small study effect test (OSDI score)

Supplemental Material 14. Meta-regression of treatment duration on OSDI score

Supplemental Material 1
Search strategy

Supplemental Material 1
Search strategy

Primary search steps:
#1. omega 3
#2. omega3
#3. omega-3 fatty acids
#4. omega-3 fatty acid
#5. omega3 fatty acids
#6. omega3 fatty acid
#7. docosahexaenoic
#8. dha
#9. eicosapentaenoic
#10. epa
#11. polyunsaturated
#12. polyunsaturated fatty acids
#13. polyunsaturated fatty acid
#14. LCPUFA*
#15. PUFA*
#16. ω-3
#17. ω3
#18. n3 fatty acid
#19. n3 fatty acids
#20. n-3 fatty acid
#21. n-3 fatty acids
#22. omega-6 fatty acids
#23. omega-6 fatty acid
#24. omega6 fatty acids
#25. omega6 fatty acid
#26. #1 OR #2 OR #3 OR #4 OR #5 OR #6 OR #7 OR #8 OR #9 OR #10 OR #11 OR #12 OR #13 OR #14 OR #15 OR #16 OR #17 OR #18 OR #19 OR #20 OR #21 OR #22 OR #23 OR #24 OR #25
#27. conjunctivitis sicca
#28. keratoconjunctivitis sicca
#29. keratitis sicca
#30. Dry Eye Disease
#31. Dry eye syndrome
#32. Dry eye
#33. cornea xerosis
#34. corneal xerosis
#35. xerophthalmia
#36. #27 OR #28 OR #29 OR #30 OR #31 OR #32 OR #33 OR #34 OR #35
#37. #26 AND #36

Final syntax in PubMed (an example):
(omega 3 OR omega3 OR omega-3 fatty acids OR omega-3 fatty acid OR omega3 fatty acids OR omega3 fatty acid OR docosahexaenoic OR dha OR eicosapentaenoic OR epa OR polyunsaturated OR polyunsaturated fatty acids OR polyunsaturated fatty acid OR LCPUFA* OR PUFA* OR ω-3 OR ω3 OR n3 fatty acid OR n3 fatty acids OR n-3 fatty acid OR n-3 fatty acids OR omega-6 fatty acids OR omega-6 fatty acid OR omega6 fatty acids OR omega6 fatty acid) AND (conjunctivitis sicca OR keratoconjunctivitis sicca OR keratitis sicca OR Dry Eye Disease OR Dry eye syndrome OR Dry eye OR cornea xerosis OR corneal xerosis OR xerophthalmia)

Supplemental Material 2

Contents and dosage of polyunsaturated fatty acids

Supplemental Material 2

Contents and dosage of polyunsaturated fatty acids

Study	Oil type	PUFA dosage (mg/day)
**Asbell et al. 2018**	Fish oil	EPA2000+DHA1000
**Barabino et al. 2003**	Medilar tablets	GLA 30+LA57
**Bhargava et al. 2013**	Not told	EPA650+DHA350
**Brignole-Baudouin et al. 2011**	Medilar (fish oil and borage oil)	427.5EPA+285+DHA+GLA15
**Chinnery et al. 2017**	fish oil	EPA1000+DHA500
**Creuzot-Garcher et al. 2011**	Nutrilarm (fish oil and borage oil)	DHA392+EPA28+ GLA82+LA126
**Creuzot-Garcher et al. 2006**	Nutrilarm (fish oil and borage oil)	DHA392+EPA28+ GLA82+LA126
**Deinema et al. 2017**	Krill oil group	EPA945+DHA510
**Kangari et al. 2013**	Fish oil and vegetable oil	EPA360+DHA240
**Kawakita et al. 2013**	Fish oil	EPA1245+DHA540
**Larmo et al. 2010**	Sea Buckthorn Oil	ALA149+ LA245
**Sheppard et al. 2013**	Hydroeye (Fish oil and black currant seed oil)	EPA126+DHA 99+ DPA39+ALA196+ GLA240+LA710+ARA3
**Wojtowicz et al. 2011**	TheraTears Nutritions (fish oil and flaxseed oil)	EPA450+DHA300+ 1000 flaxseed oil (rich in ALA)

Supplemental Material 3
Risk of bias

Supplemental Material 3

Risk of bias summary

	Random sequence generation (selection bias)	Allocation concealment (selection bias)	Blinding of participants and personnel (performance bias)	Blinding of outcome assessment (detection bias)	Incomplete outcome data (attrition bias)	Selective reporting (reporting bias)	Other bias
Asbell 2018	+	+	+	+	+	+	+
Barabino 2003	?	?	+	?	+	−	−
Bhargava 2013	+	+	+	+	+	+	+
Brignole-Baudouin 2011	+	+	+	+	+	−	+
Chinnery 2017	+	+	+	+	+	+	?
Creuzot-Garcher 2006	?	?	+	?	+	?	+
Creuzot-Garcher 2011	?	?	?	?	+	+	?
Deinema 2017	+	+	+	+	+	+	+
Kangari 2013	+	?	+	+	−	+	+
Kawakita 2013	?	?	?	?	+	−	−
Larmo 2010	?	?	+	+	−	+	−
Sheppard 2013	+	?	+	?	−	+	+
Wojtowicz 2011	?	?	+	+	+	−	−

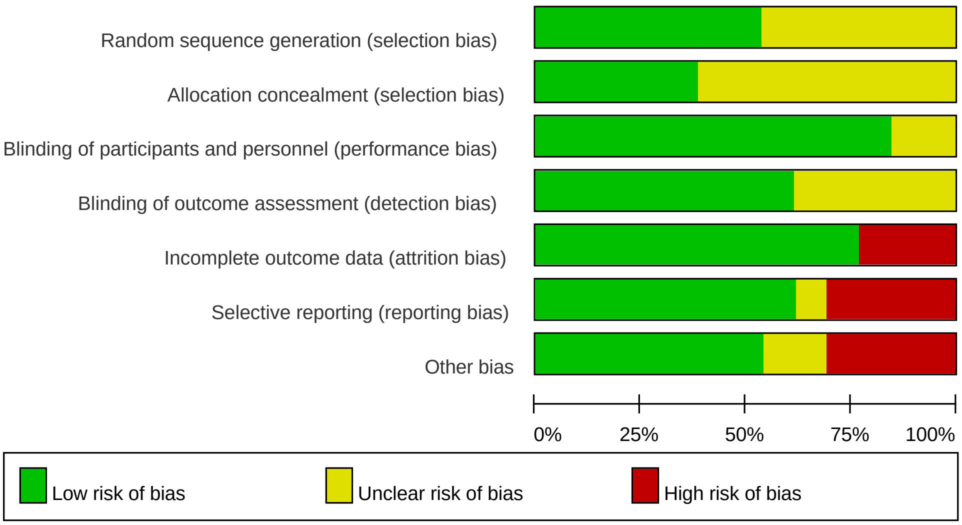

Supplemental Materials 4 to 7

Results of TBUT

Supplemental Material 4

Sensitivity analysis for high heterogeneity outcome (TBUT)

Sensitivity analysis for TBUT (Other eye medications were excluded)

Study name	Statistics with study removed						
	Point	Standard error	Variance	Lower limit	Upper limit	Z-Value	p-Value
Bhargava 2013	1.607	0.759	0.575	0.121	3.094	2.119	0.034
Deinema (k) 2017	1.503	0.509	0.259	0.506	2.500	2.954	0.003
Creuzot-Garcher 2006	2.035	0.639	0.409	0.782	3.288	3.184	0.001
Sheppard 2013	2.108	0.588	0.345	0.956	3.260	3.587	0.000
Kangari 2013	1.951	0.791	0.626	0.400	3.501	2.466	0.014
	1.799	0.565	0.319	0.691	2.907	3.182	0.001

Difference in means (95% CI) with study removed

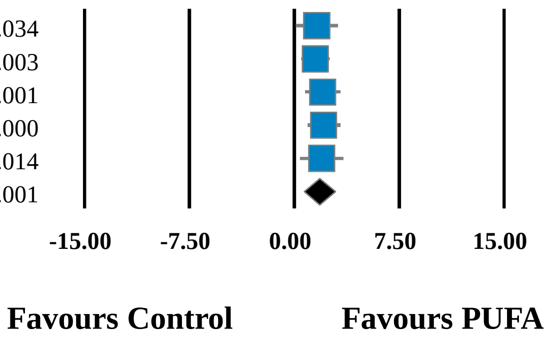

Sensitivity analysis for TBUT (Other eye medications were continued)

Study name	Statistics with study removed						
	Point	Standard error	Variance	Lower limit	Upper limit	Z-Value	p-Value
Larmo R 2010	0.142	0.118	0.014	-0.090	0.374	1.198	0.231
Larmo L 2010	0.141	0.239	0.057	-0.329	0.610	0.587	0.557
Brignole-Baudouin 2011	0.054	0.352	0.124	-0.635	0.743	0.153	0.878
Asbell 2018	-0.037	0.443	0.196	-0.905	0.831	-0.084	0.933
Creuzo-Garcher 2011	-0.022	0.232	0.054	-0.476	0.432	-0.095	0.924
	0.111	0.225	0.051	-0.331	0.553	0.492	0.622

Difference in means (95% CI) with study removed

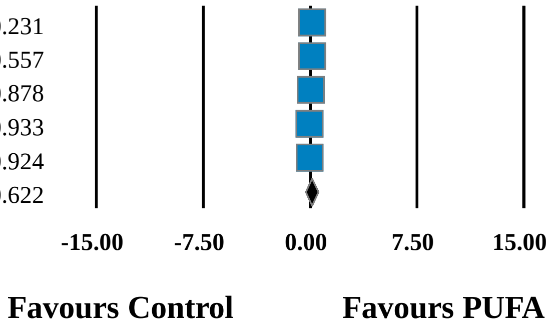

Supplemental Material 5

Small study effect test (TBUT)

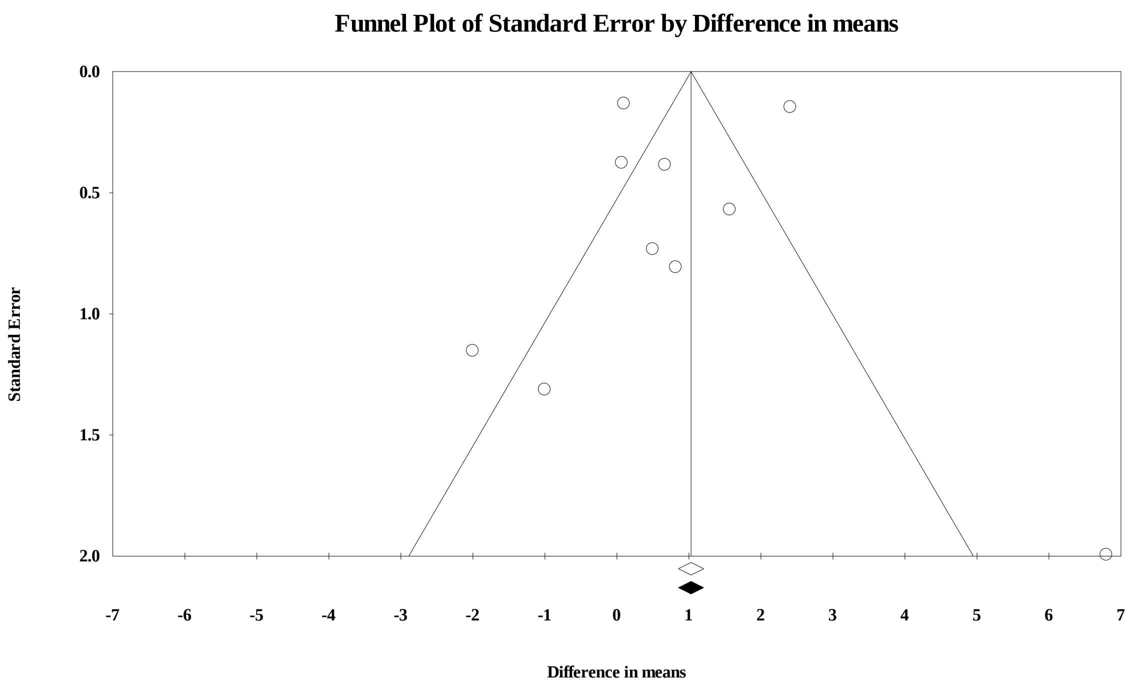

Egger's regression intercept

Intercept	-0.53142
Standard error	2.11010
95% lower limit (2-tailed)	-5.39733
95% upper limit (2-tailed)	4.33448
t-value	0.25185
df	8.00000
P-value (1-tailed)	0.40375
P-value (2-tailed)	0.80751

Supplemental Material 6

Meta-regression of treatment duration on TBUT

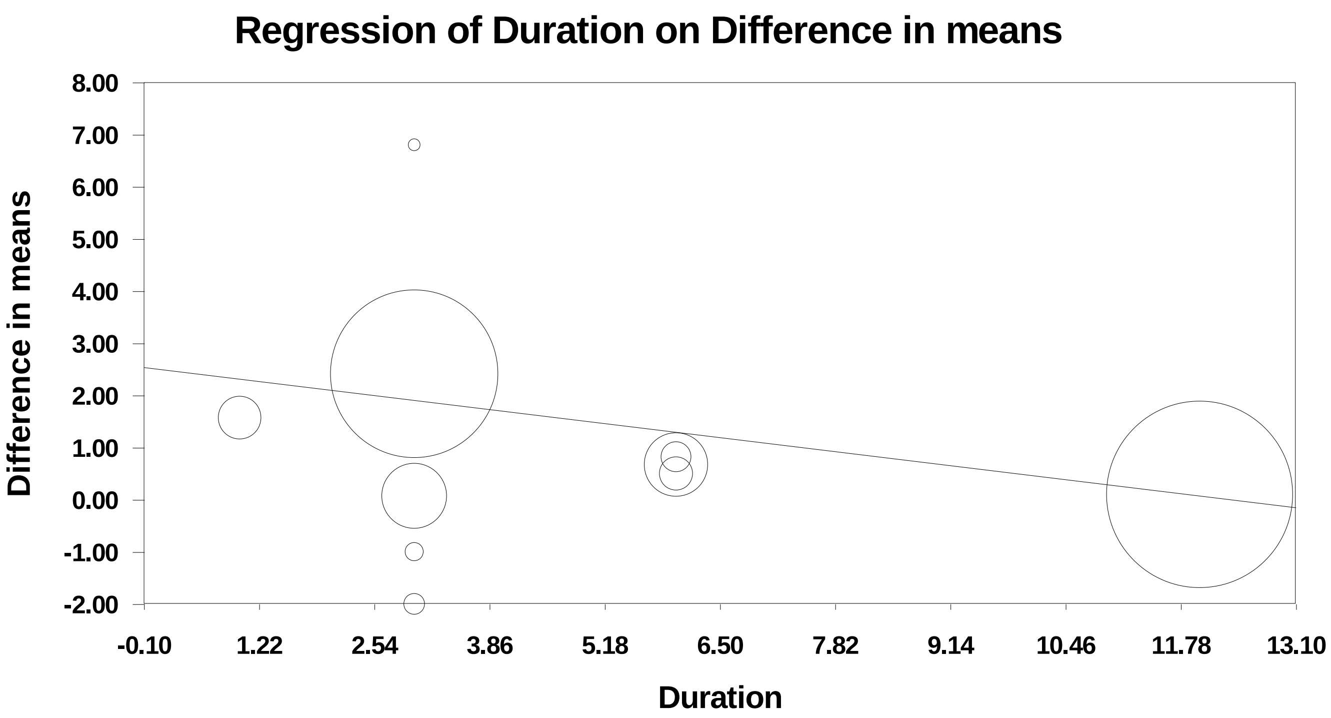

Fixed effect regression

	Point estimate	Standard error	Lower limit	Upper limit	Z-value	p-Value
Slope	-0.20368	0.02037	-0.24360	-0.16375	-9.99949	0.00000
Intercept	2.51559	0.17347	2.17560	2.85557	14.50189	0.00000
Tau-squared	1.62202					

	Q	df	p-value
Model	99.98978	1.00000	0.00000
Residual	63.70172	8.00000	0.00000
Total	163.69150	9.00000	0.00000

Supplemental Material 7

Meta-regression of single-eye data on TBUT

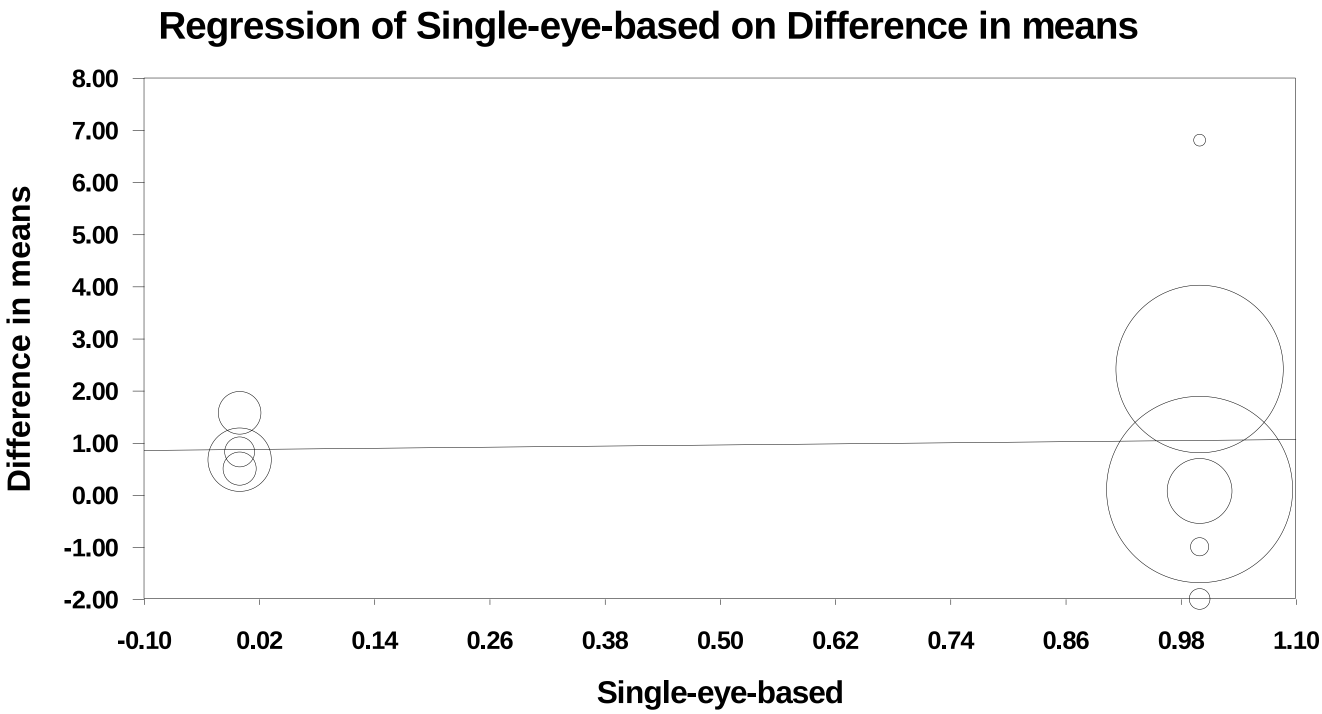

Fixed effect regression

	Point estimate	Standard error	Lower limit	Upper limit	Z-value	p-Value
**Slope**	0.17454	0.29110	-0.39600	0.74508	0.59960	0.54877
**Intercept**	0.87341	0.27530	0.33384	1.41298	3.17262	0.00151
**Tau-squared**	2.16381					

	Q	df	p-value
**Model**	0.35952	1.00000	0.54877
**Residual**	163.33198	8.00000	0.00000
**Total**	163.69150	9.00000	0.00000

Supplemental Material 8 to 11

Results of Schirmer test score

Supplemental Material 8

Small study effect test (Schirmer's test)

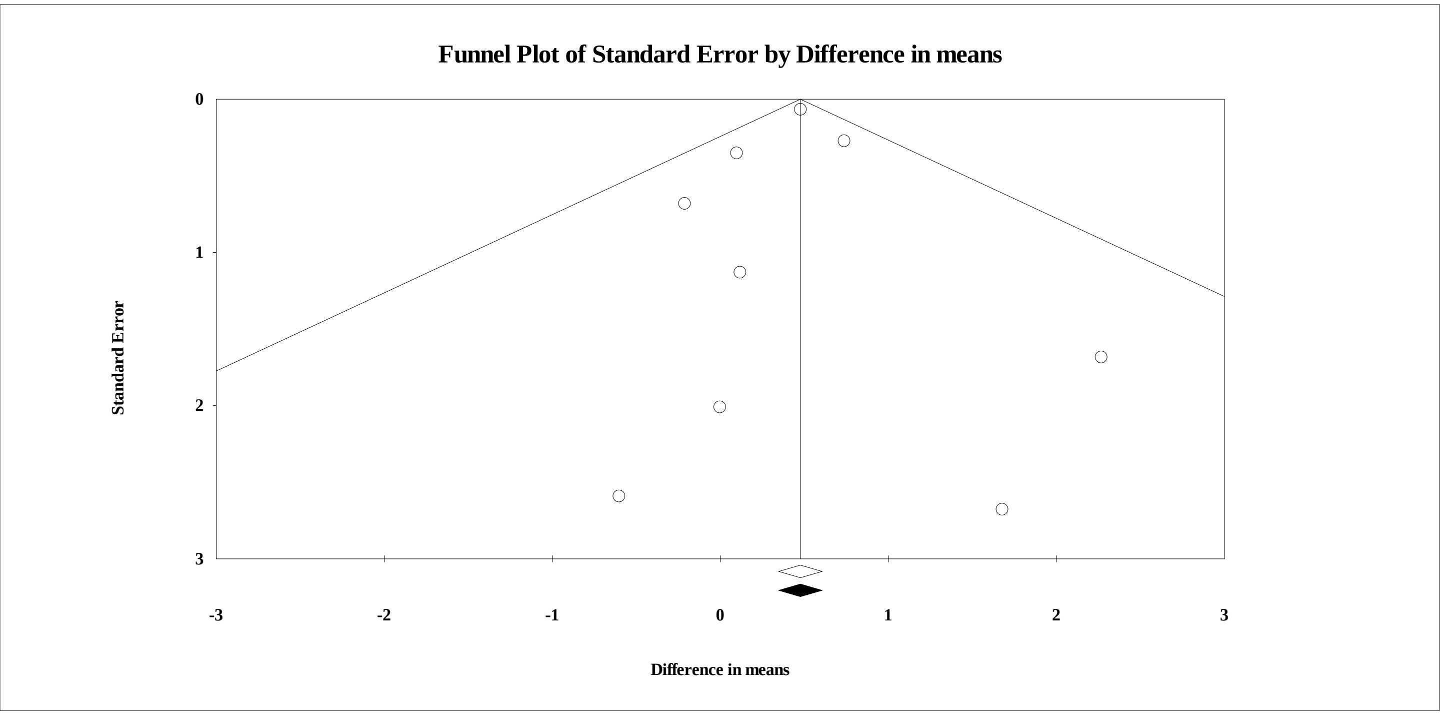

Egger's regression intercept

Intercept	-0.08101
Standard error	0.32572
95% lower limit (2-tailed)	-0.85121
95% upper limit (2-tailed)	0.68919
t-value	0.24871
df	7.00000
P-value (1-tailed)	0.40536
P-value (2-tailed)	0.81072

Supplemental Material 9

Meta-regression of treatment duration on Schirmer's test score

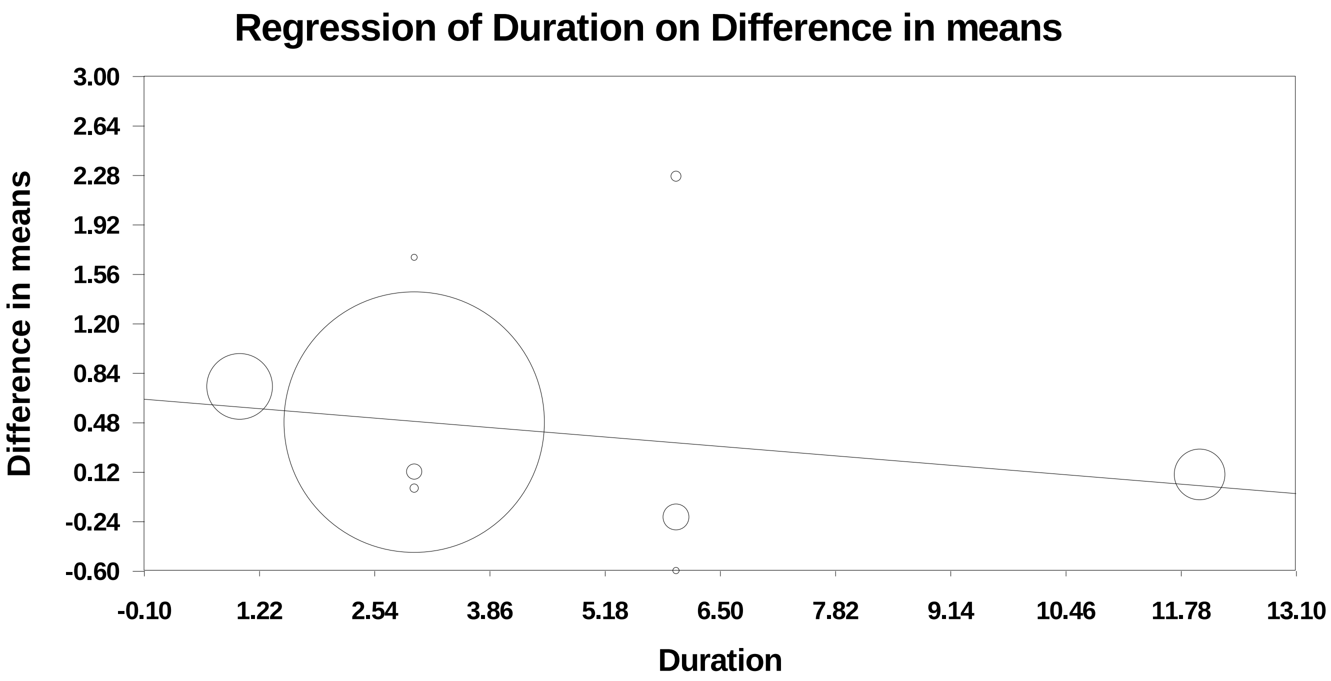

Fixed effect regression

	Point estimate	Standard error	Lower limit	Upper limit	Z-value	p-Value
Slope	-0.05205	0.03756	-0.12567	0.02157	-1.38581	0.16580
Intercept	0.64459	0.13817	0.37377	0.91540	4.66509	0.00000
Tau-squared	0.00000					

	Q	df	p-value
Model	1.92048	1.00000	0.16580
Residual	2.79089	7.00000	0.90365
Total	4.71137	8.00000	0.78793

Supplemental Material 10

Meta-regression of single-eye data on Schirmer's test score

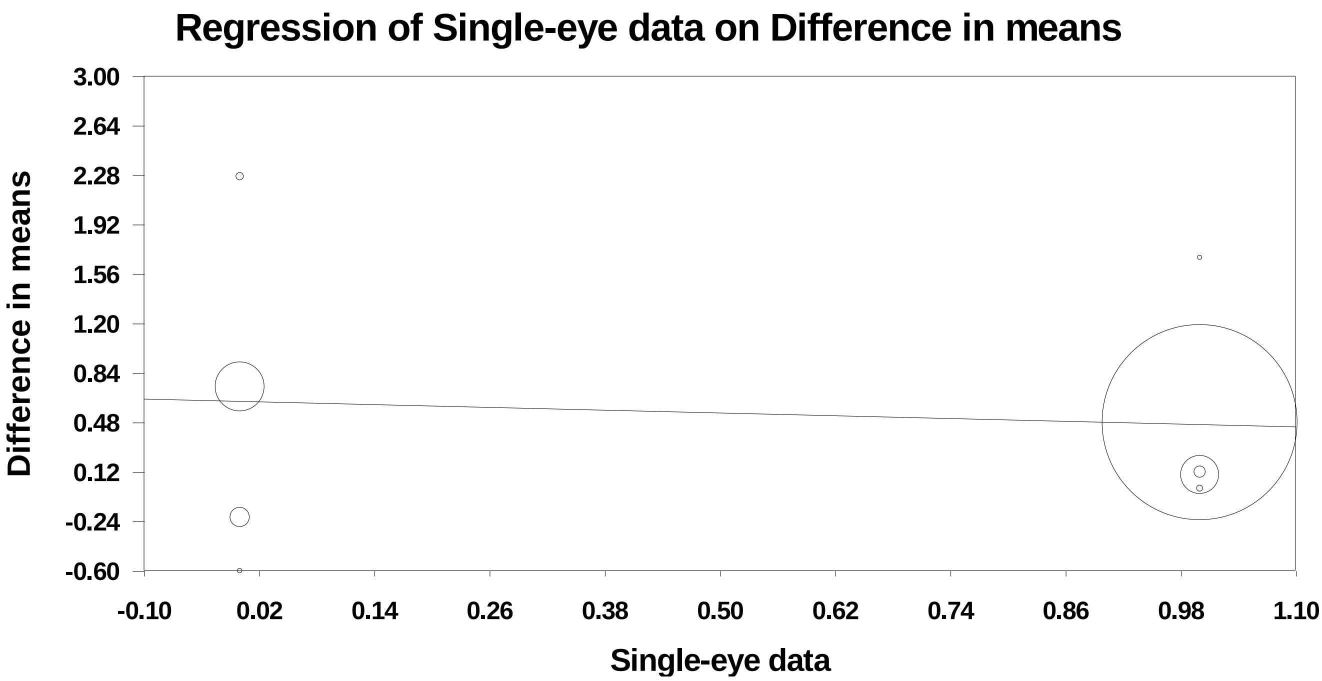

Fixed effect regression

	Point estimate	Standard error	Lower limit	Upper limit	Z-value	p-Value
**Slope**	-0.16838	0.26058	-0.67911	0.34235	-0.64617	0.51817
**Intercept**	0.63314	0.25144	0.14032	1.12596	2.51803	0.01180
**Tau-squared**	0.00000					

	Q	df	p-value
**Model**	0.41753	1.00000	0.51817
**Residual**	4.29384	7.00000	0.74538
**Total**	4.71137	8.00000	0.78793

Supplemental Material 11

Meta-regression of anesthesia on Schirmer's test score

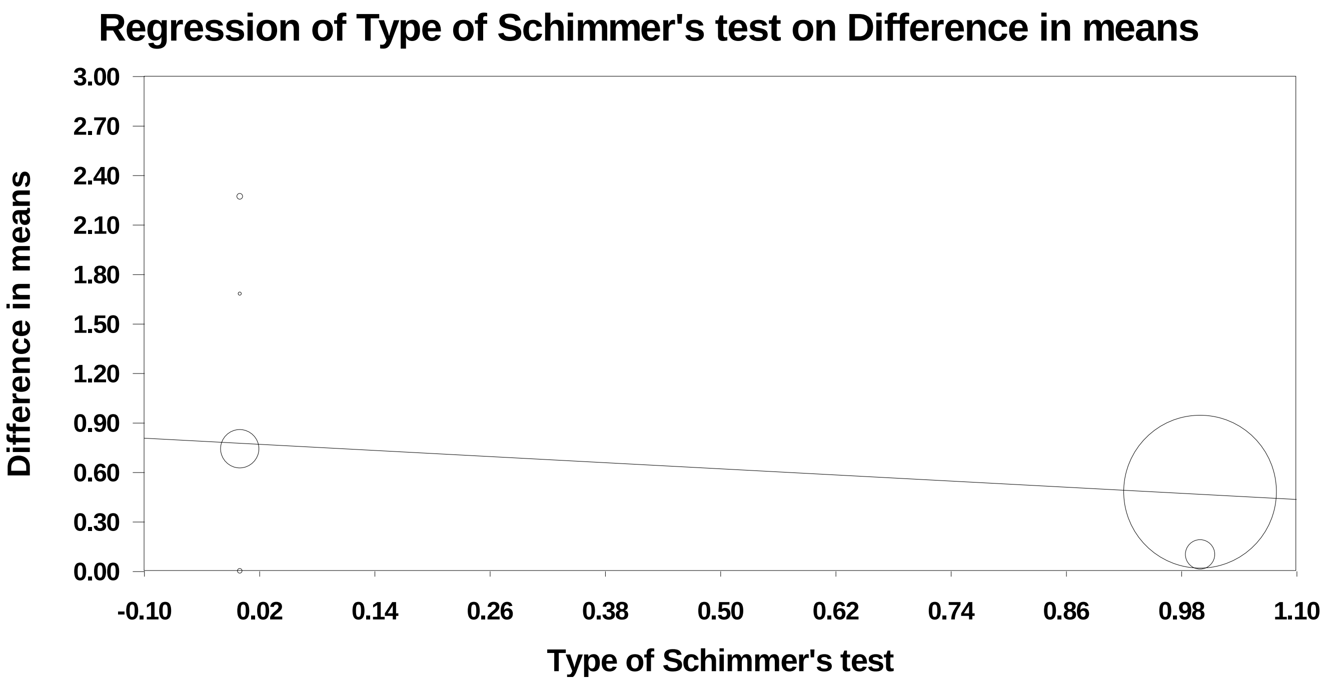

Fixed effect regression

	Point estimate	Standard error	Lower limit	Upper limit	Z-value	p-Value
Slope	-0.30913	0.27668	-0.85141	0.23314	-1.11730	0.26386
Intercept	0.77490	0.26804	0.24956	1.30025	2.89101	0.00384
Tau-squared	0.00000					

	Q	df	p-value
Model	1.24837	1.00000	0.26386
Residual	2.16996	4.00000	0.70453
Total	3.41833	5.00000	0.63578

Supplemental Material 12

Result of Osmolarity

Supplemental Material 12

Small study effect test (Osmolarity)

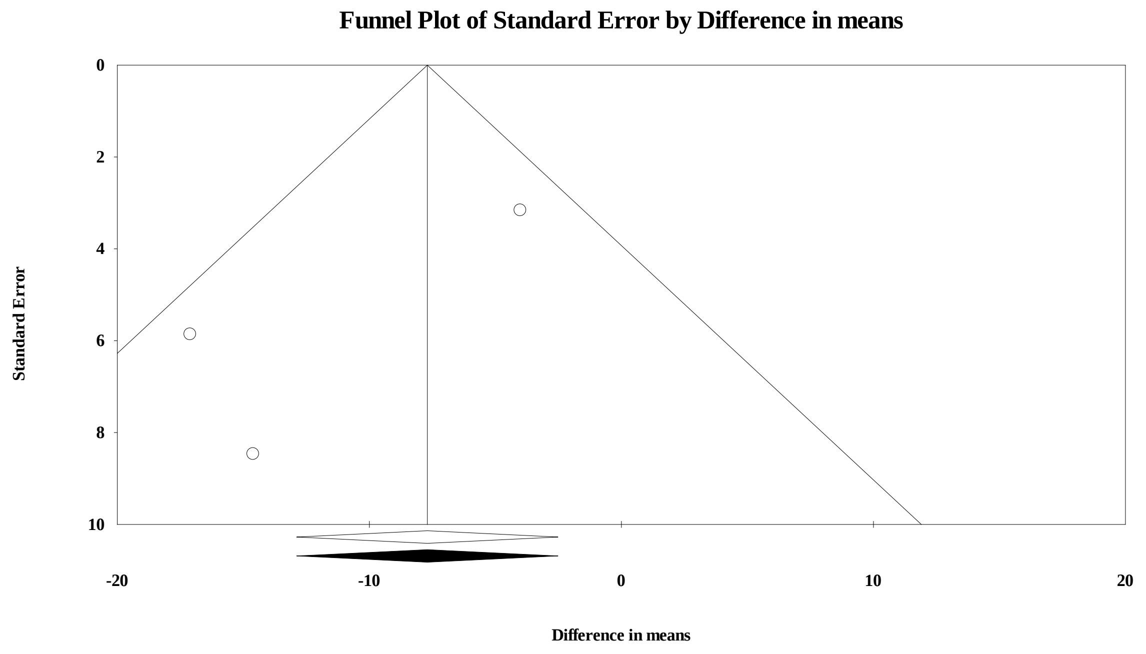

Egger's regression intercept

Intercept	-2.82615
Standard error	1.55884
95% lower limit (2-tailed)	-22.63309
95% upper limit (2-tailed)	16.98079
t-value	1.81298
df	1.00000
P-value (1-tailed)	0.16045
P-value (2-tailed)	0.32089

Supplemental Material 13 to 14

Results of OSDI

Supplemental Material 13

Small study effect test (OSDI score)

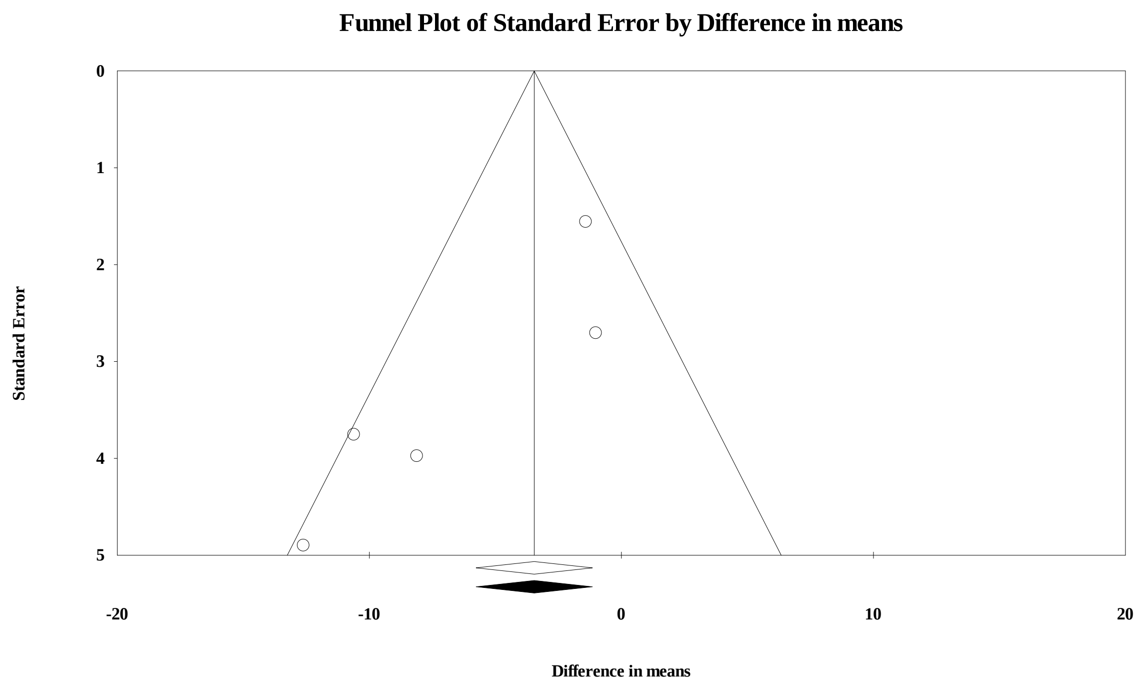

Egger's regression intercept

Intercept	-3.18359
Standard error	0.96981
95% lower limit (2-tailed)	-6.26995
95% upper limit (2-tailed)	-0.09723
t-value	3.28271
df	3.00000
P-value (1-tailed)	0.02317
P-value (2-tailed)	0.04633

Begg and Mazumdar rank correlation

**Kendall's S statistic (P-Q)**	-6.00000

Kendall's tau without continuity correction

Tau	-0.60000
z-value for tau	1.46969
P-value (1-tailed)	0.07082
P-value (2-tailed)	0.14164

Kendall's tau with continuity correction

Tau	-0.50000
z-value for tau	1.22474
P-value (1-tailed)	0.11034
P-value (2-tailed)	0.22067

Supplemental Material 14

Meta-regression of treatment duration on OSDI score

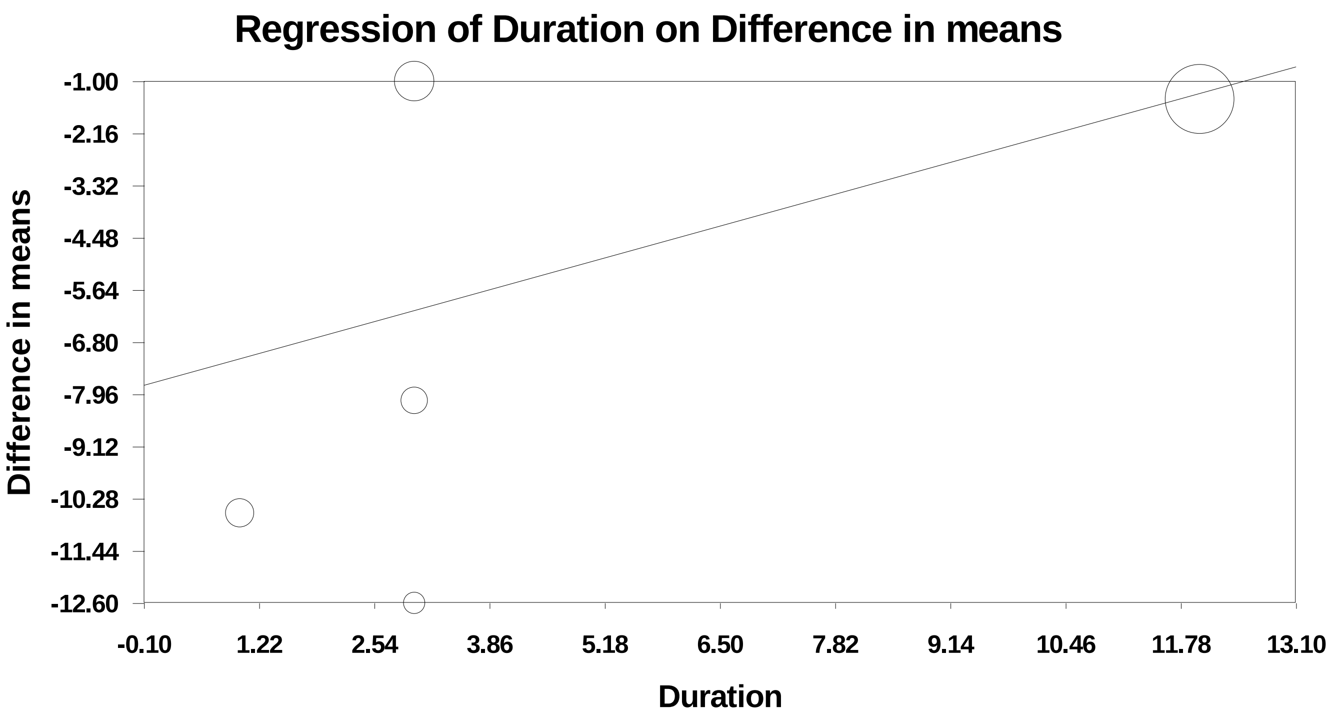

Fixed effect regression

	Point estimate	Standard error	Lower limit	Upper limit	Z-value	p-Value
Slope	0.53640	0.24957	0.04725	1.02555	2.14928	0.03161
Intercept	-7.70513	2.29968	-12.21241	-3.19784	-3.35052	0.00081
Tau-squared	15.27261					

	Q	df	p-value
Model	4.61939	1.00000	0.03161
Residual	6.39341	3.00000	0.09396
Total	11.01280	4.00000	0.02642
